# Should I Get Screened for Sleeping Sickness? A Qualitative Study in Kasai Province, Democratic Republic of Congo

**DOI:** 10.1371/journal.pntd.0001467

**Published:** 2012-01-17

**Authors:** Alain Mpanya, David Hendrickx, Mimy Vuna, Albert Kanyinda, Crispin Lumbala, Valéry Tshilombo, Patrick Mitashi, Oscar Luboya, Victor Kande, Marleen Boelaert, Pierre Lefèvre, Pascal Lutumba

**Affiliations:** 1 Programme National de Lutte contre la Trypanosomiase Humaine Africain (PNLTHA), Kinshasa, Democratic Republic of Congo; 2 Institute of Tropical Medicine, Antwerp, Belgium; 3 Institut Nationale de Recherche Biomédicale, Kinshasa, Democratic Republic of Congo; 4 Bureau Diocesain des Œuvres Médicales, Mbuji-mayi, Democratic Republic of Congo; 5 Université de Mbuji-mayi, Mbuji-mayi, Democratic Republic of Congo; 6 Université de Kinshasa, Kinshasa, Democratic Republic of Congo; 7 Université de Lubumbashi, Lubumbashi, Democratic Republic of Congo; IRD/CIRDES, Burkina Faso

## Abstract

**Background:**

Control of human African trypanosomiasis (sleeping sickness) in the Democratic Republic of Congo is based on mass population active screening by mobile teams. Although generally considered a successful strategy, the community participation rates in these screening activities and ensuing treatment remain low in the Kasai-Oriental province. A better understanding of the reasons behind this observation is necessary to improve regional control activities.

**Methods:**

Thirteen focus group discussions were held in five health zones of the Kasai-Oriental province to gain insights in the regional perceptions regarding sleeping sickness and the national control programme's activities.

**Principal Findings:**

Sleeping sickness is well known among the population and is considered a serious and life-threatening disease. The disease is acknowledged to have severe implications for the individual (e.g., persistence of manic periods and trembling hands, even after treatment), at the family level (e.g., income loss, conflicts, separations) and for communities (e.g., disruption of community life and activities). Several important barriers to screening and treatment were identified. Fear of drug toxicity, lack of confidentiality during screening procedures, financial barriers and a lack of communication between the mobile teams and local communities were described. Additionally, a number of regionally accepted prohibitions related to sleeping sickness treatment were described that were found to be a strong impediment to disease screening and treatment. These prohibitions, which do not seem to have a rational basis, have far-reaching socio-economic repercussions and severely restrict the participation in day-to-day life.

**Conclusions/Significance:**

A mobile screening calendar more adapted to the local conditions with more respect for privacy, the use of less toxic drugs, and a better understanding of the origin as well as better communication about the prohibitions related to treatment would facilitate higher participation rates among the Kasai-Oriental population in sleeping sickness screening and treatment activities organized by the national HAT control programme.

## Author Summary

Active screening strategies are common disease control interventions in the context of poor and remote rural communities with no direct access to healthcare facilities. For such activities to be as effective as possible, it is necessary that they are well adapted to local socio-economic and cultural settings. Our aim was to gain insight into the barriers communities in the Kasai-Oriental province of the Democratic Republic of Congo experience in relation to their participation in active screening activities for African sleeping sickness. Participation rates seem to be especially low in this province compared to other endemic regions in the country. We found several important factors to be in play, a number of which could be addressed by adapting the operational procedures of the mobile teams that perform the active screening activities (e.g., improved confidentiality during the screening procedure). However, more profound considerations were found in the form of regional beliefs related to the treatment of the disease. Although not based on rational grounds, these prohibitions seem to pose a significant barrier in a person's decision to seek diagnosis and treatment. A better understanding of these prohibitions and their origin could lead to improved participation rates for sleeping sickness screening in Kasai-Oriental.

## Introduction

**Figure 1:**
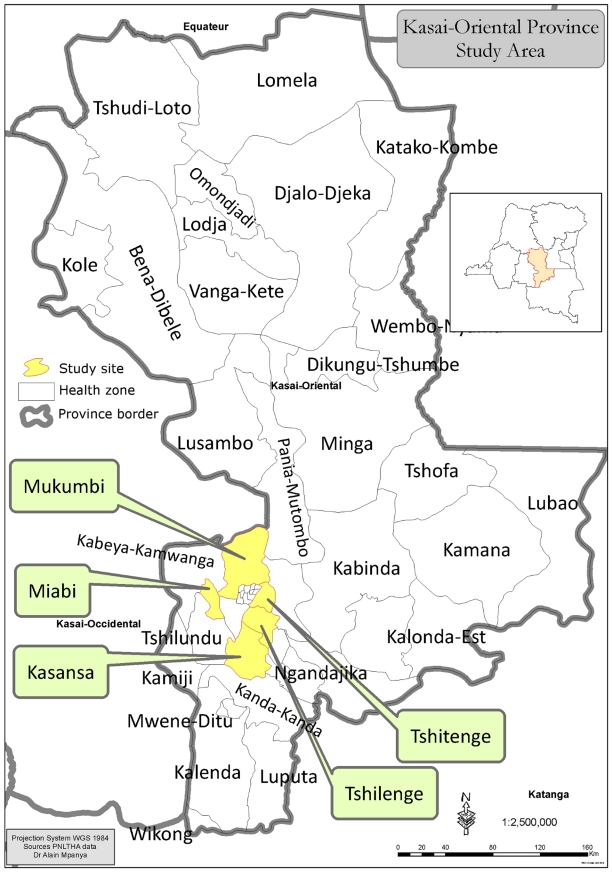
Geographic locations of the health zones included in the study.

Human African Trypanosomiasis (HAT) or African sleeping sickness is a parasitic disease unique to sub-Saharan Africa. It is caused by protozoa of the *Trypanosoma* Genus of which the species *T. brucei gambiense* and *T. brucei rhodesiense* cause disease in humans. According to WHO figures [1], [2] the disease is present in 36 sub-Saharan countries, where 60 million people are at risk of which less than 4 million are under surveillance. In 2006 the annual number of new cases was estimated to be between 50,000 and 70,000, but only a fraction of that number of cases is reported. Between 1998 and 2009 the number of annually reported HAT cases has dropped from 37,991 to 9,878 [2], [3]. *T. brucei gambiense* causes the chronic form of the disease which is endemic in central and western Africa, while the acute form caused by *T. brucei rhodesiense* is found in East-Africa [4].

HAT control strategies are based on early case detection and treatment and vector control [1], [5]–[7]. Active screening strategies conducted by mobile teams which travel from village to village have substantially lowered the case load in several African countries, most notably in Uganda and Sudan [8]–[10]. In the Democratic Republic of Congo (DRC), where *T. brucei gambiense* is endemic, this control strategy has led to a considerable decrease in case numbers throughout the whole country, albeit with a large variability among the endemic provinces. In the North Equator and Kinshasa provinces a significant decrease in prevalence was observed. However, the number of cases detected in two other provinces, Bandundu and Kasai-Oriental, has remained unabated notwithstanding an increased screening effort [11], [12]. Many factors could explain this intervariability in HAT prevalence among provinces which otherwise are all subject to the same HAT control strategy. Amongst others, such factors could be related to a low coverage of the population at risk, low community participation rates in active screening and treatment activities, or an inefficient management and coordination of the mobile teams. Treatment failure could also play an important role, as studies have shown significant treatment failure rates across the country's provinces, from 5 to 10% in Bandundu, to 25% in North Equator, and possibly as high as 50% in Kasai-Oriental [13], [14]. In 2007 adherence to treatment in DRC was at 91.6% [15], with very little variation between endemic provinces. A recent analysis of the operational effectiveness of HAT screening and treatment activities in Kasai-Oriental (stagnation of infection rates) and North Equator (decrease of infection rates) in 1998, 2001 and 2005 by Lumbala (unpublished, manuscript in preparation) shows that low community participation rates in active screening activities and coverage of the population at risk could well explain the differences between these two provinces. While issues related to the coverage of the population at risk lie with the management and coordination of the national control programme's activities (availability of resources, control strategy), community participation rates reflect the level of health service utilization which can be influenced by various social, economical and cultural factors.

It is thought that the low participation rate of the rural population in the active screening activities is one of the main reasons for the continued HAT transmission in Bandundu and Kasai-Oriental. In a study on the effectiveness of active population screening and treatment for sleeping sickness in DRC, Robays et al [12] observed that the overall mean participation rate in the provinces of Equateur, Bandundu and Kasai-Oriental was 74%. However, important variability of the attendance rates between villages, between mobile teams and between provinces were observed. In Bandundu and Kasai-Oriental, participation rates as low as 64% and 50% were respectively found.

The reasons for this low rate of community participation in screening activities in these two provinces has been little investigated, although a better understanding of relevant cultural and socio-economic barriers could significantly improve the effectiveness of HAT control programmes. Robays et al [16] showed for example that in DRC's Bandundu province the fear of drug toxicity, financial barriers and the lack of confidentiality during screening were the most important obstacles for participation in the HAT campaign in that province. As a result of those findings, the DRC's HAT control program abolished the nominal user fee for screening. Although this fee was minimal, it still presented a hurdle for large families with no or little income. These findings might be considered indicative for other provinces of DRC, but care should be taken not to generalise as socio-economic and cultural contexts are very heterogeneous across the country. Therefore, this study aims to document in a qualitative manner the economic and socio-cultural factors which may influence community participation in active HAT screening and treatment activities in the Kasai-Oriental province, where the disease puts not only rural communities at risk but also a large group of workers who are engaged in diamond mining activities. Additionally, as is illustrated by the difference in local languages (Tshiluba in Kasai-Oriental, Kikongo in Bandundu), the Kasai-Oriental and Bandundu provinces differ distinctly on a cultural level, which may influence local disease perceptions and health seeking behaviour habits.

## Methods

### Study area

We performed a transversal descriptive qualitative study using the focus group discussion (FGD) technique in five health zones in the Kasai-Oriental province of DRC: Miabi, Tshilenge, Tshitengue, Kasansa and Mukumbi. Figure 1 shows the geographic locations of these health zones.

These health zones were selected on the basis of their location in a historic and currently active focus of HAT near the Kasai-Oriental capital of Bakwanga (Kalelu- Lubilashi) which represents almost 70% of the total number of reported cases in the province [17]. Table 1 shows the HAT prevalence data for all five health zones in 2005 and 2007. Along with the region's linguistic and cultural characteristics, the socio-economic setting in Kasai-Oriental is very diverse due to the presence of a diamond industry which employs many workers who live in encampments surrounding the diamond mines. In Kasai-Oriental the main populations at risk are diggers working in the diamond mines and farmers, who represent the most active groups in the population.

**Table 1:** Prevalence data for the health zones included in the study.

Health Zone	Prevalence 2005	Prevalence 2007
Kasansa	0.17%	0.09%
Miabi	0.18%	0.07%
Mukumbi	0.77%	0.32%
Tshilenge	0.32%	0.19%
Tshitengue	0.08%	0.30%

Source: 2005 and 2007 annual reports of the national control programme division of Kasai-Oriental.

### Focus group discussions

The data collection took place in January 2008. Thirteen FGDs were conducted. The focus groups were divided along three categories: gender, geographic characteristics (i.e. worker camp or village) and health zone. Table 2 shows the characteristics of each of the FGDs performed in this study.

**Table 2:** Characteristics of the focus group discussions.

FGD nr	Geographic characteristic	Number of participants	Gender	Health zone
1	Diamond mine camp	7	Men	Tshitengue
2	Diamond mine camp	9	Women	Tshitengue
3	Village	8	Men	Kansansa
4	Village	6	Women	Kansansa
5	Village	9	Men	Miabi
6	Village	7	Men	Miabi
7	Village	9	Women	Miabi
8	Village	8	Women	Miabi
9	Village	9	Men	Mukumbi
10	Diamond mine camp	7	Men	Mukumbi
11	Diamond mine camp	7	Mixed	Mukumbi
12	Village	8	Women	Tshilenge
13	Village	6	Men	Tshilenge

The number of FGDs conducted in the different health zones reflects the “richness” of the information found in those health zones. We continued to hold focus group discussions in a particular health zone until data saturation was reached and no new information was coming out of them. The FGDs were stratified by gender to ensure the homogeneity of the groups and to promote openness during the discussions, as women in general do not speak freely in front of men in Kasai-Oriental. One of the focus groups in Mukumbi was mixed because we were not able to identify a minimum of six women willing to participate in an FGD in that specific location.

The question guide used in the FGDs was pre-tested and fine tuned in two focus group discussions performed in a health zone not included in the study area. The quality of these discussions was evaluated with the research team before finalizing the question guide. The following topics were covered: general knowledge of the disease; community practices regarding the disease; the community's attitude towards the mobile teams; the community's participation in screening and treatment activities; and community's expectations regarding HAT control services.

The FGDs numbered six to nine participants who were invited by the principal investigator (A.M.). They were selected at random based on their availability and whether they gave consent for their participation. Two exclusion criteria were used: (i) participants had to be resident of the local community for at least 2 years (a definition also used by the national HAT control programme in their population surveillance data); (ii) community leaders such as teachers, village chiefs and priests were excluded in order to avoid them dominating the discussion dynamics. The FGDs took place in a hut assigned by the community chief for this purpose. The average duration of the discussions was 45 minutes. They were held in the local language, Tshiluba. A local doctor and nurse, both native Tshiluba speakers, were trained to moderate and observe the FGDs. They switched roles for each discussion. Both had previously never been involved in the screening activities of the national HAT control programme. Their training, which was conducted by A.M., consisted of a two-day course during which they were briefed on the study objectives and taught how to conduct FGDs. Each discussion was evaluated and discussed post-hoc by A.M. and the two assistants. A.M. attended all the FGDs to supervise the process. All discussions were fully recorded on a digital audio recorder.

### Data analysis

The FGDs were transcribed and translated into French by the two research assistants, who took turns in both tasks. A secretary prepared the transcripts in Microsoft Word. A.M. revised all the transcripts prior to analysis. QSR Nvivo8 was used to support the data analysis and identify trends in the data. This software allows researchers to organize and analyze complex and unstructured datasets by fragmenting and categorizing data whilst keeping a link with the source documents (transcripts of the FGDs in this case). The analysis itself is an inductive process which allows themes to emerge from the data. These themes are coded into categories which are continuously refined throughout the analysis. Finally, relationships between categories are created and inferences are made. The analysis and its process were discussed with the co-investigators.

The FGDs were coded, each element of the analysis representing an intervention by a focus group discussion participant. The codes were developed progressively and in an inductive manner, allowing relevant themes to emerge from the data. The coding book was discussed and refined with the co-investigators to assure the significance of the analysis.

### Ethical statement

The study protocol was approved by the thematic HAT institutional review board (IRB) in DR Congo and the IRB of the Institute of Tropical Medicine, Antwerp, Belgium. Local community authorities were asked for permission to perform the study in their villages. Focus group discussion participants were informed about the voluntary nature of their participation. Permission for tape recording the conversations was requested prior to starting each of the focus group discussions. Anonymity of the participants was guaranteed and no personal details were recorded. Oral consent was obtained and audio recorded before the start of each focus group discussion. Oral consent was preferred since regional literacy levels are low. This procedure for consent was approved by the IRB.

## Results

In this section we will present the various aspects of community perceptions related to HAT which were elaborated during the FGDs. Data is presented in the form of quotes from the FGDs. The quotes used in this section are illustrative of the sentiments reflected in the FGDs and were selected on the basis of their aptness and informative quality.

### General knowledge and perceived symptoms

In general, sleeping sickness is well known in the region and is considered an affliction which has been around for many generations. In the local tongue it is referred to as ‘disama dya tulu’, which literally translates to ‘sleeping sickness’. *“Sleeping sickness has been around for ages. We have heard people talking about it since our childhood. It is an ancient disease.”* [FG10] Nevertheless, whilst many referred to specific cases from their direct environment during the discussions -*“I know sick people. My mother has the disease and so does my sister.”* [FG4]- , not all participants had personally been confronted with the disease in the past. *“Well, I can't really say as I have never seen the disease. A typical example of a case? I haven't seen one yet.”* [FG1] A number of people said they did not have any ‘scientific knowledge’ of the disease and were not completely at ease with the medical rational approach adopted by the health workers of the national control programme. *“I know sleeping sickness exists, but the knowledge about how to avoid it is given by doctors who ask people to avoid this or that disease in this or that way.”* [FG10]

When discussing the symptoms which they relate to the disease, behavioural problems, sleep, tiredness, fever and headaches were all commonly referred to in the FGDs. Behavioural problems and the linked personality changes on the one hand, and irregular sleeping patterns on the other, were considered the most tell-tale signs of sleeping sickness. Women seemed to be more knowledgeable about the symptoms and their relation to the disease than men. This could be explained by the women's traditional role as caregiver. When a family member becomes sick, it is generally the mother who accompanies them to the health services and who is briefed by the health staff about the disease and how to take care of the patient. Women are also more likely to participate in the disease screening activities and thus to interact with health workers, a trend which was also observed in other studies [18], [19]. The symptoms which are typical for the early stage of the disease, such as fever, headaches and tiredness, seem to be perceived less in the worker camps surrounding the diamond mines than in the villages. When the disease is in its late neurological stage the affected person is considered to be unaware of his state. Rather, it is his entourage which identifies him as abnormal and unable to take care of himself. *“When a person has this disease, she is not able to reason for herself. Only those who are at her side can do it in her place.”* [FG5]

### Perceived severity and social consequences

Sleeping sickness is perceived as a severe illness since many people are affected by it and many patients die. *“Many people have the disease, because we have seen with our own eyes how many people have died of it. We can't give an exact number, but many people have the sleeping sickness.”* [FG4] The consequences of sleeping sickness are numerous and very visible within the communities, adding to the perceived severity.

The disease has serious repercussions on the patient, the family and the community. The high case fatality rate and the iatrogenic deaths induced by the toxic drug melarsoprol are very well known. Moreover, a large number of participants in our FGDs pointed towards the neuropsychiatric sequelae in those surviving, such as lunacy and trembling hands, which do not always regress after treatment. *“This disease turns people into idiots. In order to control him, he needs to be restrained by force, even if he doesn't agree with that. He doesn't want a child to come near him. If it does happen, he might kill the child. It's the same with tall people. Afterwards, when he has been treated, his spirit never returns to normal. He remains confused and doesn't know how to do things.”* [FG13] It is considered shameful to be affected by the disease and stigmatisation is common. This does however not imply a rejection by the community, but rather signifies a shift of the patient's place in the community. In other words, the social role of the patient in the community changes, together with the expectations, rights and obligations which go along with that role.

At the level of the family the repercussions are for one part socio-economic since when a family breadwinner becomes ill he no longer is able to work and provide for the family's needs. *“The concern is that the sick person could be working to support the family. But now with him being sick, all those who depended on him share in his misfortune.”* [FG5] The disease puts many additional strains on family ties and marriages. When the man is sick for example, the woman is often obliged to leave the house in order to avoid sexual relations with her husband, which is locally considered to be strictly forbidden for actual or recovering HAT patients. If it is the woman who is sick, the man in general goes in search of another partner. This can have drastic consequences for the family unit, as illustrated in the following quote: *“Women can also get the disease, in which case the man doesn't wait. He takes another woman into his house and puts his partner out on the street together with the children, as they might be sorcerers, so they are banished. We see them every day. We call them ‘the children of the market’”* [FG6]. In some instances a case of HAT can also lead to family conflicts caused by the search for a potential sorcerer considered to be at the origin of the disease. *“Sometimes they say that the sick person's mother, paternal aunt or uncle is at the source of the disease. They seem to forget that the tsé-tsé fly is where the disease comes from.”* [FG13] On the community level, an increase of HAT cases can have an impact on the general development of the village. *“One can say that many people mainly work in the fields. When someone becomes sick, she no longer has the strength to cultivate the fields. That has a negative impact on the village because she no longer produces food for the village.”* [FG5] In extreme cases this can even lead to an implosion of the local community. *“When many people, or everybody, becomes sick, no work is done anymore. Many have become idiots, others behave like madmen. The village is dead.”* [FG4] Such social and economic consequences can be very far reaching and eventually lead to forced migration to other villages.

### Perceived aetiology

In general, the vector of African sleeping sickness and its role in disease transmission is well known in the communities. The tsetse fly was elaborated upon in all FGDs and is locally known under various names: dibudu, bibuiba and kabwibwibwa. *“The fly bites, she leaves behind this disease. She can be found in the villages, in the forests and near water. This insect that we call ‘dibubu’”* [FG2] However, several other causes or modes of transmission were also stated. For example, traditional beliefs and sorcery were sometimes referred to when the spread of African sleeping sickness was discussed. *“When the adults of the village address solemn words against this disease, it diminishes. So it is provoked by the realm of darkness.”* [FG6] Some women talked about the role of the amaranth—a green plant cultivated in the region—and pigs as a source of the diseases. *“When we arrived here, they told us that the amaranth gives people the sleeping sickness. When a person eats the amaranth, she will get the sleeping sickness.”* [FG8] *“I have already heard that the disease comes from the pigs here in the village. They say that bad pigs carry this disease.”* [FG7] Such beliefs are likely grounded in the indirect role amaranths and pigs might play in the diseases, transmission, since tsetse flies seem to be more abundant in and around crop fields and animal pens.

Other discussants thought that the members of the mobile teams played an important role in spreading the disease, transmitting the disease while they perform the screening. *“Observing how those who have the disease suffer, the population thinks that the nurses of the team carry the disease and transmit it to you the moment you stand in front of them.”* [FG5] Finally, although rarely mentioned in the focus group discussions, contagion was also elaborated as a means by which the disease spreads. *“Because it is a contagious disease. If we eat together with him, we might get contaminated.”* [FG1]

### Prevention

In general, the communities talk about vector control activities as the most important way of prevention. This further reflects their general understanding of the tsetse fly's role in the transmission of the disease. *“We take our machetes to cut down the trees, palms and others. Bibuibua will be scared and take flight.”* [FG3] *“How the disease can be avoided? By pouring medicine in the rivers and placing traps for the bibuibua.”* [FG3] *“That person should be educated and told to wear white clothes when she goes into the jungle.”* [FG6] On the other hand, there is a feeling that prevention is in essence impossible because villagers cannot abandon their livelihood activities, which take place in locations, such as the fields, where the tsetse fly is found. *“There is no way to avoid contact since when we go to the fields the flies bite us.”* [FG6] The importance of raising awareness in communities is also referred to as an efficient way of preventing infections. The mobile teams are seen as an important channel in this respect. When they arrive in a village and before they start the screening activities, the nurses of the team give a lesson on how to avoid the disease. *“They explain us how we can avoid getting the disease, how the disease enters the village and why we should avoid shabbiness around our houses.”* [FG3]

### Barriers to screening

The low attendance rates at the screening activities organised by the national HAT control programme pose a significant problem. When discussing the reasons for this observation during the FGDs, six main barriers were identified: giving priority to occupational activities; the toxicity of the drugs used in treatment; distrust towards the nurses of the mobile teams; fear of lumbar punctures; fear of unsolicited HIV/AIDS tests; and the lack of confidentiality during the screening procedure itself.

The population wakes early in the morning to leave for the diamond mines or crop fields. The mobile teams generally arrive in the villages later in the morning, with little or no prior notice, after most of the working populace have already left. Furthermore, people tend to avoid screening as long as they consider themselves to be healthy. Why risk being diagnosed and having to give up your livelihood activities for a prolonged period of time for treatment if you do not feel sick? *“The people are afraid. Everyone reasons that if they catch me with sleeping sickness, I will no longer be able to do all my work. My activities won't be able to take place anymore. So it is better not to be tested as long as I don't feel sick. Once I do, then I go to the doctors. They will take care of me.”* [FG12] This quote not only refers to the inability to work during treatment, but also for a significant period after treatment. The root of this logic lies in the regionally accepted notion that one must adhere to a number of prohibitions for six months after having been treated for HAT. Labouring is such a prohibition and is therefore considered to be strictly forbidden during the six-month rest period. These prohibitions are further elaborated below in the section on barriers to treatment.

Drug toxicity is generally considered an important barrier to participation in the active screening activities. Also in this context people do not feel compelled to participate in the screening process as long as they feel healthy and consider the risks of screening to outweigh the benefits. *“Many people have died, even the one who has only been injected once. You see him die, and he wasn't even sick. People are frightened and think: ‘If I have myself tested, I might be giving up my activities for nothing and I might even die.’*” [FG3]

As was previously indicated when discussing the perceived aetiology of HAT, there seems to be a degree of distrust from the community towards the nurses of the mobile teams. In several FGDs the latter were suspected of injecting the disease during the screening procedure. This idea arises from the perception that even people considered to be in good health are regularly diagnosed with the disease by the mobile teams. *“The people refuse to have themselves tested because the nurses are going to inject them with the disease. They leave the insect of sleep behind through their injections, making us sick.”* [FG7] Another example of this distrust which was voiced in several FGDs is the belief that an HIV test is part of the HAT screening procedure. There is a fear that one's potentially positive HIV status could be disclosed to the community, a risk people are not willing to take given the consequences. If someone is identified as HIV-positive, he or she becomes the focus of mockery and runs the risk of being rejected by the community. *“They refuse to present themselves to the doctors because it is possible they are caught with AIDS. It is not unlikely as AIDS is present here.”* [FG9]

The screening procedure itself was also criticized in the discussions. Especially the lack of confidentiality during the screening activities was considered to be an important issue. The procedure mostly takes place on a village square in plain sight of all those queuing up for the screening. It is considered embarrassing to be tested in public. Furthermore, people are afraid their disease status would become public knowledge. *“Me, I feel shame about the possibility of being caught with this disease in public because I would be mocked. Therefore, I don't present myself for these tests.”* [FG5] The lumbar puncture in open air, which is part of the screening procedure, is also considered as a significant barrier. *“Others are afraid of the syringe as it hurts in the spine. We are afraid of it.”* [FG5]

### Barriers to treatment

Cases confirmed by the mobile teams are referred to HAT treatment centres. However, also here several important barriers can be identified. The toxicity of the drugs, the financial inaccessibility, the prohibitions related to the treatment, the lacking geographic accessibility of the treatment centres, the sense of feeling healthy notwithstanding a positive diagnosis, and the fear of the regular lumbar punctures which are performed during the follow-up of the treatment are all elements which influence one's decision to seek treatment after positive diagnosis by the mobile teams.

Treatment of sleeping sickness in the region is free, but presents significant indirect costs to the patient and his escorts, mainly related to the travel to treatment centres on one hand, and nourishment on the other. *“When I was caught with the disease, I left the village and left to Gandajika. I didn't have any family there, I was accompanied by my wife. When we ran out of food, we had to go back to our village for food before returning to the treatment center. This was a huge problem. That is why offering treatment in centres far away from the village brings along many difficulties to the sick. How will they get to the centre? How do they feed themselves? How can they be monitored by their relatives?”* [FG13] This opinion, especially regarding the financial repercussions, was shared by many, though financial factors seemed to be less of a barrier for those living in the encampments surrounding the diamond mines.

One's perceived health state is not only a factor in deciding whether to participate in the screening activities, but also for the next step, when a confirmed HAT case is referred to a treatment centre. Some people diagnosed with sleeping sickness simply do not feel sick and do not see the need to go through the long and painful process of treatment. *“They had diagnosed her with this disease. Up to today she has decided not to receive treatment and she still is as she always has been, in good health. So the people make mistakes with their tests, and therefore we can't have them done.”* [FG5]

Of special interest are a number of generally accepted prohibitions linked to the treatment of sleeping sickness. Patients are expected to adhere to these prohibitions during a six month resting period after treatment. The following prohibitions, illustrated by the quotes in Table 3, were elaborated upon during the FGDs: no walking in the sun; no warm meals and hot spices; no alcohol consumption; no smoking; no heavy labour; and no sexual relations. The social and economic implications of these prohibitions form important barriers to HAT screening and treatment activities. The patient's chance of survival and the probability of making a full recovery are perceived as being directly linked to the degree to which the patients stick to the prohibitions. Treatment failure and other complications are blamed on the individual, reasoning they brought it upon themselves by not adhering to the prohibitions. Because of their importance, there is a strong element of social control involved, as the patient's entourage is mobilised to help him stick to the prohibitions. The communities are very much aware of these prohibitions. They were a recurring theme in most FGDs. Although the cited six month rest period is an existing guideline from the national HAT control programme [20], the specific prohibitions mentioned in the FGDs are not.

**Table 3:** Citations illustrating the prohibitions linked to the resting period after treatment.

*“ The major problem regarding sleeping sickness is the fact that people are asked not to have sexual relations with their wife, even when they have sexual desires. When the patient has gone through the treatment and has returned home, during his ensuing rest period, he doesn't respect the prohibition and he dies.” [FG6]*
*“A patient in his rest period should not eat hot food, only cold food. He should not walk in the sun, drink alcohol, smoke, work or have sexual relations.” [FG10]*
*“He may not eat hot things, stay under the sun, or stay in places where there is smoke. He must avoid things that have poison, and one should make sure he does not drink alcohol. A person must be near at all times to keep an eye on him, to make sure he avoids all these things.” [FG10]*

## Discussion

African sleeping sickness is well known and recognised as a serious disease in the communities of Kasai-Oriental province. In general the symptoms, vector, and treatment procedures for sleeping sickness are well known amongst the population. Fear of drug toxicity, lack of confidentiality during screening procedures and financial barriers were all elaborated upon in the FGDs as primary reasons for non-participation in the active screening activities organised by the national control programme.

These findings are in line with a similar study conducted in the province of Bandundu [16], although two additional important barriers came forward in our focus group study.

The first is the apparent incompatibility between the itineraries of the mobile screening teams and the population's livelihood activities. By the time the mobile screening teams arrive in the village, many workers have already set out to the diamond mines or crop fields. Part of this problem could be due to a lack of communication between the mobile teams and the communities. Improved planning of the screening activities to assure compatibility with the local population's habits would also be important in this respect. Factors such as daily routines and seasonal variations in the communities' activities should therefore be taken into account.

A second barrier which seems to be of particularly high importance in Kasai-Oriental is the generally accepted belief that a HAT patient must adhere to a number of prohibitions for a period of six months after receiving treatment. These hold important social and economic implications. For example, it is forbidden to perform heavy labour for the first six months after treatment. This prohibition makes it nearly impossible to make a living during this period and signifies an important loss of income. Additionally, by implying significant restrictions on participation in everyday activities, the prohibitions also lead to a degree of social exclusion and put a considerable amount of pressure on family relationships. Strong social control regarding the prohibitions is in place and victim blaming is common. When a person becomes sick, suffers a relapse or dies during the rest period, this is considered to be the result of non-adherence to the prohibitions. Given the profound impact of the six month rest period and the accompanying prohibitions, their observed role as a barrier to active HAT screening and treatment activities in Kasai-Oriental is not surprising. Even when a person is diagnosed with HAT they will refuse to go to treatment centers up until the time they become severely ill. For the national HAT control programme, a good treatment is one that is administered within 10 days after diagnosis. The problem is however that part of the population refuses to even participate in active screening activities, as they prefer not to know with certitude whether they have sleeping sickness, thus ensuring they are able to avoid treatment and all the prohibitions linked therewith. Although similar prohibitions were reported in the Bandundu study by Robays et al [16], in our study they seem to be a more profound element in the motivations of the community's actions as they constituted a recurring theme in almost all focus group discussions.

Notwithstanding the important role the prohibitions seem to take up in the communities' perception regarding HAT, their precise origin remains unclear. However, the fact that the prohibitions do not seem to have a medical basis does not make them any less significant as a barrier to HAT screening and treatment. On the contrary, a better understanding of their origin would be a first step in a process which could lead to a significantly higher participation rate in the national programme's screening activities in Kasai-Oriental. Further in-depth research is therefore necessary to document them. The focus of such research should not only be on the communities, but should also investigate the possible roles of the health workers in HAT treatment centers and the mobile teams.

The prohibitions related to nutrition may lie in traditional naturalistic or holistic beliefs about illness and health. Maintaining a form of natural balance is a central concept in such theories. Many Hispanic, African, Arab and Asian cultures incorporate elements of this basic idea in their understanding of disease. The Yin/Yang theory used in Asian cultures is a well-know example of such an approach. Another relatively common holistic framework used in traditional understanding of health and illness is based on a hot/cold dichotomy [21]–[23]. Some diseases are considered hot, whilst others are considered cold. The same dichotomy is applied to beverages, foods, herbs and medicines. Classification in either category is not necessarily based on physical characteristics of the item, but is more often according to their perceived effects on the body or their association with natural elements. There is a strong element of flexibility and subjectivity at play in the process of hot/cold classification. Regional and cultural variations in classification are therefore common. However, what is considered to be universal in this mode of thought is the practice of perceiving disease as a hot-cold imbalance which must be restored in order for the sick person to be cured. A similar logic might be at work behind the prohibitions which forbid warm meals, hot spices and possibly alcohol. If sleeping sickness is regarded as a hot disease, hot foods and beverages would be considered to upset the patient's hot/cold balance after treatment rather than allowing it to be fully restored. This imbalance, if not resolved, may then be perceived to lead to complications, relapse or even death.

It appears from our FGDs that patients often remain with neurological and psychiatric sequelae. Sleeping sickness is a chronic disease that evolves in two stages. Stage 1, or the hemolymphatic stage, is characterized by nonspecific symptoms and can even remain asymptomatic [24]. The treatment for early stage HAT is of low toxicity and recovery is complete. Unfortunately at this stage the patient often does not seek care as symptoms are mild or absent. When the patient does seek care, the non-specific symptoms are often confounded with malaria—which is also endemic in Kasai-Oriental—leading to misdiagnosis and a delay in receiving correct treatment. Stage 2 HAT is characterized by an infection of the nervous system which leads to neurological and psychiatric disorders. It is often only at this stage that a patient seeks care. The first-line treatment for second- stage HAT in DRC at the time of this study was Melarsoprol, a toxic organic compound of arsenic. Treatment with Melarsoprol can cause severe side-effects such as arsenical encephalopathy in 5 to 10% of cases, which can lead to neurological damage and death [25], [26]. In Kasai-Oriental an important population at risk consists of diamond diggers, an active portion of the population. These workers are very concerned about their professional activities to the point of ignoring their own health-related problems. As long as they have the strength to continue working in the diamond mines, they will not seek medical care, even if they are ill. Only when they become severely sick and are no longer able to work do they go to health centers. They therefore often present with well-advanced HAT infections. Treatment at such a late stage is problematic and mostly does not improve the neurological and psychiatric sequelae caused by the disease, even after cure. Melarsoprol treatment failure is high in Kasai-Oriental and—as was shown in this study—is often accredited to non-compliance to the 6 month resting period and the many prohibitions linked therewith. Patients are blamed and are themselves held responsible if they do not make a full recovery after treatment. Ultimately, the fear of possibly failing to comply with these prohibitions becomes a barrier to active HAT screening and treatment in itself.

The toxicity of the drugs used to treat HAT remains an important barrier in a person's decision to participate in the national programme's active screening activities. The adverse effects of Melarsoprol are well documented [26], and the communities are well aware of the risks associated to its use in treatment. In November 2009 the WHO started rolling out a new nifurtimox-eflornithine combination therapy (NECT) in DRC. NECT has shown very high cure rates and low adverse effect rates. Furthermore, it is relatively easy to administer, requiring only fourteen infusions over the course of ten days. Although many challenges remain [27], NECT has the potential to hugely improve the level of HAT care which is delivered to communities. However, it would be prudent to consider that it might take some time before the implementation of NECT positively influences the participation rates in the national programme's active screening activities. After all, perceptions and beliefs such as the fear of drug toxicity are deep-rooted and do not change with the wind. The legacy of melarsoprol will therefore most likely remain a barrier for some time to come. If NECT is to live up to its full potential, it is clear that communication and sustained community participatory approaches have important roles to play in the activities of national HAT control programmes, not just in the Democratic Republic of Congo, but also in all other HAT endemic countries. Additional socio-anthropological studies similar to the one reported in this paper could offer valuable insights in this respect.

## References

[1] WHO (1998). La Trypanosomiase Africaine: lute et surveillance..

[2] WHO (2006). Human African trypanosomiasis (sleeping sickness): epidemiological update.. Wkly Epidemiol Rec.

[3] Simarro PP, Cecchi G, Paone M, Franco JR, Diarra A (2010). The Atlas of human African trypanosomiasis: a contribution to global mapping of neglected tropical diseases.. Int J Health Geogr.

[4] Burke J (2000). La trypanosomiase humaine africaine.

[5] Burke J, Janssens PG, Kivits M, Vuylsteke J (1992). Les trypanosomiasis Africaines.. Médecine et Hygiène en Afrique Centrale de 1885 à nos jours.

[6] Simarro PP, Sima FO, Mir M, Mateo MJ, Roche J (1991). [Control of human African trypanosomiasis in Luba in equatorial Guinea:evaluation of three methods].. Bull World Health Organ.

[7] Stanghellini A, Dumas M, Bouteille B, Buguet A (1999). Prophylactic strategies in human African trypanosomiasis.. Progress in human African trypanosomiasis, sleeping sickness.

[8] Moore A, Richer M, Enrile M, Losio E, Roberts J (1999). Resurgence of sleeping sickness in Tambura County, Sudan.. Am J Trop Med Hyg.

[9] Moore A, Richer M (2001). Re-emergence of epidemic sleeping sickness in southern Sudan.. Trop Med Int Health.

[10] Paquet C, Castilla J, Mbulamberi D, Beaulieu MF, Gastellu Etchegorry MG (1995). [Trypanosomiasis from Trypanosoma brucei gambiense in the center of north-west Uganda. Evaluation of 5 years of control (1987–1991)].. Bull Soc Pathol Exot.

[11] Lutumba P, Robays J, Miaka mia BC, Mesu VK, Molisho D (2005). Trypanosomiasis control, Democratic Republic of Congo, 1993–2003.. Emerg Infect Dis.

[12] Robays J, Bilengue MM, Van der Stuyft P, Boelaert M (2004). The effectiveness of active population screening and treatment for sleeping sickness control in the Democratic Republic of Congo.. Trop Med Int Health.

[13] Robays J, Nyamowala G, Sese C, Kande V, Lutumba P (2008). High Failure Rates of Melarsoprol for Sleeping Sickness, Democratic Republic of Congo.. Emerg Infect Dis.

[14] Pépin J, Bokelo M (2005). Trypanosomiasis Relapse after Melarsoprol Therapy, Democratic Republic of Congo, 1982–2001.. Emerg Infect Dis.

[15] PNLTHA (2007).

[16] Robays J, Lefevre P, Lutumba P, Lubanza S, Kande Betu KM (2007). Drug toxicity and cost as barriers to community participation in HAT control in the Democratic Republic of Congo.. Trop Med Int Health.

[17] CTB, PNLTHA (2004).

[18] Asonganyi T, Ade S (1994). Sleeping sickness in Cameroon.. Journal Camerounais de Médecine.

[19] Pepin J, Mpia B, Iloasebe M (2002). Trypanosoma brucei gambiense African trypanosomiasis: differences between men and women in severity of disease and response to treatment.. Trans R Soc Trop Med Hyg.

[20] Bureau Central de la Trypanosomiase (1995).

[21] Bailey EJ (2000). Medical Anthropology and African American Health.

[22] Beardsworth A, Keil T (1997). Sociology on the Menu: An Invitation to the Study of Food and Society.

[23] Manderson L (1987). Hot-cold food and medical theories: overview and introduction.. Soc Sci Med.

[24] Burri C, Brun R, Cook GC, Zumla A (2003). Human African Trypanosomiasis.. Mansons's Tropical Diseases 21st ed.

[25] Blum J, Nkunku S, Burri C (2001). Clinical description of encephalopathic syndromes and risk factors for their occurrence and outcome during melarsoprol treatment of human African trypanosomiasis.. Trop Med Int Health.

[26] Barrett MP, Boykin DW, Brun R, Tidwell RR (2007). Human African trypanosomiasis: pharmacological re-engagement with a neglected disease.. Br J Pharmacol.

[27] Yun O, Priotto G, Tong J, Flevaud L, Chappuis F (2010). NECT is next: implementing the new drug combination therapy for Trypanosoma brucei gambiense sleeping sickness.. PLoS Negl Trop Dis.

